# Polymer Composites with Quantum Dots as Potential Electrode Materials for Supercapacitors Application: A Review

**DOI:** 10.3390/polym14051053

**Published:** 2022-03-07

**Authors:** Himadri Tanaya Das, Paritosh Barai, Swapnamoy Dutta, Nigamananda Das, Payaswini Das, Madhusudan Roy, Md. Alauddin, Hasi Rani Barai

**Affiliations:** 1 Centre of Excellence for Advanced Materials and Applications, Utkal University, Bhubaneswar 751004, Odisha, India; dasn.chem@utkaluniversity.ac.in; 2 Department of Biochemistry and Molecular Biology, Primeasia University, Dhaka 1213, Bangladesh; paritoshbarai9@gmail.com; 3 CEITEC BUT, Brno University of Technology, Purkynova 123, 612 00 Brno, Czech Republic; swapnamoy12@gmail.com; 4 CSIR-Institute of Minerals and Materials Technology, Bhubaneswar 751013, Odisha, India; payaswinidas@gmail.com; 5 Department of Computer Science and Engineering, University of Science and Technology Chittagong, Chattogram 4202, Bangladesh; madhusudan@ustc.ac.bd; 6 Department of Theoretical and Computational Chemistry, University of Dhaka, Dhaka 1000, Bangladesh; alauddin1982@du.ac.bd; 7 Department of Mechanical Engineering, Yeungnam University, Gyeongsan 38541, Korea

**Keywords:** polymers, Quantum Dots, nanocomposites, electrodes, electrolytes, supercapacitors, energy storage

## Abstract

Owing to the nanometer size range, Quantum Dots (QDs) have exhibited unique physical and chemical properties which are favourable for different applications. Especially, due to their quantum confinement effect, excellent optoelectronic characteristics is been observed. This considerable progress has not only uplifted the singular usage of QDs, but also encouraged to prepare various hybrid materials to achieve superior efficiency by eliminating certain shortcomings. Such issues can be overcome by compositing QDs with polymers. Via employing polymer composite with QDs (PQDs) for supercapacitor applications, adequate conductivity, stability, excellent energy density, and better specific capacitance is been achieved which we have elaborately discussed in this review. Researchers have already explored various types of polymer nanocomposite with different QDs such as carbonaceous QDs, transition metal oxide/sulphide QDs etc. as electrode material for supercapacitor application. Synthesis, application outcome, benefits, and drawbacks of these are explained to portray a better understanding. From the existing studies it is clearly confirmed that with using PQDs electrical conductivity, electrochemical reactivity, and the charge accumulation on the surface have prominently been improved which effected the fabricated supercapacitor device performance. More comprehensive fundamentals and observations are explained in the current review which indicates their promising scopes in upcoming times.

## 1. Introduction

Energy generation and distribution became a point of concern for the upcoming days as the conventional fossil fuel seems to be almost exhausted. This unfortunately stirred chaos has made the peoples of the last few decades to move towards exploring the renewable energy sources like wind, solar, biofuels etc., which have posed significant ability to replace the dependency of the conventional energy sources or precisely the use of coal and oil. Not only the scarcity of the coal and oil like natural sources, but also their resulting damaging impact on the environment is another potential reason to get inclined towards the nontoxic or very lesser toxic renewable energy sources. Various experiments have already exhibited their very negligible or no threats to the nature, hence globally they are being considered for permanent usage to make the daily energy consumption more precise and safer, and hence to cease further unrestricted expansion of global warming. To use these energy sources in more effective way, constructing prominent energy storage became a necessary option. In this regard electrochemical energy storage devices such as battery, supercapacitors (SCs) and fuel cell are been considered and widely explored and as of now noteworthy development has been observed which is positively impacting every sector of technology as well as human lives. Due to having excellent power density and superior device performance, several studies have suggested extensive utilization SCs in energy storage applications. However, in recent days batteries hold much better reputation in industrial scale usage as the energy density performance of batteries are much reliable than SCs. But batteries cost of production is higher than the SCs and SCs cost can be further reduced if potential cost effective electrodes are chosen for charge storage. Even the batteries performance has satisfactory cycles still not enough to meet the demand of advance electronics whereas SCs have a very long-term cycle life. Besides that, major drawbacks of batteries are the lack of safety, low flexibility and heavy mass. All these issues can be overcome easily by flexible small compact SCs and also safety major can be taken care by implementing solid-state or semi-solid or gel-polymer electrolyte in SCs [1]. In general, SCs are divided into several categories such as EDLC SCs, pseudo-SCs, faradaic-SCs or hybrid SCs. Usually the carbonaceous materials are considered as EDLCs materials [2,3]. Where the metal oxides/chalcogenide exhibit the pseudocapacitor behaviour. Further, battery-type metal oxides/chalcogenides undergo faradaic reactions for charge storage process [4,5].

Amalgamation of new-age nanotechnology and utilization of favourable nanomaterials of SCs have created enormous encouraging scopes and developments which raised promising future prospects for electronic technologies. These approaches will hopefully shape the next generation electronics to be more compact and economical as well. However, studies are still focusing on their working mechanisms rather than only advancing their components as harmonizing between both is the key to higher efficiency. Mainly, the SCs are comprised of two working electrodes with a separator positioned in between them to isolate them and along with that a fitting electrolyte is been employed to make a complete working setup. In several studies, these components like electrolyte and electrodes are been varied and explored to a greater extent. For instance, various ranges of materials of different size and morphologies are already been investigated as electrode materials in last few decades and several promising results are been reported [6]. Though in recent studies, restrictions are been applied on the utilization of materials which have toxic impact on the environment [7]. Nonetheless, these limitations didn’t actually stop the actual advancement, but made the approaches more precise in terms of evaluating the material and analysing their efficacious impact. Recent research has also concentrated on the utilization of everyday life generated waste materials or abandoned wastages to employ for energy application purposes. These waste derived materials are using for different components in the SCs. Through these approaches, not only the scientific fields are getting adequate advantages but also is a prominent way to reduce growing issues to handle waste materials. Regarding this, biomass derived materials have exhibited great opportunities and even utilizing plastic materials for energy storage devices is been taken into consideration in recent research [8,9].

Owing to the favourable characteristics like higher surface to volume ratio, enriched surface area, superior concentration of edge atoms, quantum confinement effect, easy solubility in non-aqueous and aqueous solvents, functionalization and easy doping conditions, Quantum Dots (QDs) are been considered for various range of applications like photovoltaics, bio-sensing, light-emitting diodes, bio-imaging, batteries, fuel cells etc. [10,11]. Moreover, research findings have also shown their viability in coalescing with other nanomaterials to develop better workable composites for application purposes. Commonly QDs are zero dimensional materials having diameter of 2–10 nm and are preferred in several applications as the described size range have evidently shown stable quantum confinement effect with efficient results and controllable physical and chemical properties [12,13]. The major noticeable factor is the enhanced surface area of QDs which is adequately beneficial for SCs application. This surface area criteria easily improves the high charge storage ability of the SC materials. Moreover, the higher concentration of edge atoms, high density of active sites, larger surface to volume ratio, and size effects assists in transportation or movement activity of ions (especially adsorption or desorption of ions). Hence, usage of different QDs is very favourable for SCs application. In this review, we will elaborate about various types of QDs along with their benefits and drawbacks along with mentioning their several tested setups of SCs. In addition, we will also focus about different synthesis techniques used in studies to prepare QDs of different sizes and morphologies.

In recent studies, utilization of polymers, especially conductive polymers have attracted much attention. Along with delivering excellent specific energy, they have exhibited optimum pseudocapacitive nature which resulted in faster redox reaction and hence superior electrochemical activity [14,15]. Researchers have utilized conductive polymers of different sizes or dimensions and different morphologies (nanoparticles, nanosheets, nanofibers) in energy storage and conversion applications [16]. However, limitations like low mechanical strength, inadequate solubility, shrinkage, and swelling during charge-discharge cycles have restricted their capacitive behaviour. In order, to achieve further improvements studies have recommended compositing carbonaceous compounds with these polymer materials. This composite preparation approach has resulted in improved conductivity, thermal stability, and intermolecular chain reaction and hence significant changes like better rate capability and enhanced capacitance observed in SC device performance [17].

## 2. Importance of Various QDs and Polymers as Electrode Material in Supercapacitors

In order to search for suitable material and to comprehend their individual performances, vast range of materials (includes 0D, 1D, 2D and 3D) are been investigated using different supercapacitor (SCs) setups along with the study of their particular electrochemical activity [18]. In addition, various composite or hybrid materials are also been tested to achieve improvement in device outcome [19]. Performance parameters such as cycling, high specific power, charging–discharging rate etc. are been mainly considered as evaluating factors. Besides, aspects like size or morphology of the materials, chemical and physical stability, electrical conductivity, specific surface area, corrosion resistance, and surface wettability are also found to be considerable parameters in order to fabricate better performing as well as safer devices [20]. For an example, electrode materials consist of smaller pores exhibits higher capacitance but in few cases have shown lesser power density, on the other hand, in the electrode material the distribution of meso or micropores seemed to be a key factor in mass and ion transport, hence superior electrochemical activity is been observed. In recent times, eco-friendly device preparation are also been focused due to rising environmental concerns [21]. So, selection of appropriate nanomaterial for supercapacitor electrodes is been a vital task which seem to have major influence in the overall activity of supercapacitor. However, not only the electrodes but also electrolytes, separators etc. also have significant contribution in the SCs performance.

Carbonaceous compounds, conducting polymers, and transition metal oxides/hydroxides are the materials, which are frequently been, employed in SCs electrodes. Owing to easy synthesis, better surface area, inexpensiveness, and lesser hazardous impact on nature carbonaceous materials are regarded as one of the best candidates for SCs or other energy storage devices. Carbon-based materials also have shown adequate conductivity, stability (chemical and thermal) and symmetrical galvanostatic charge–discharge profile which makes it more attractive for SCs application. Majorly, activated carbon, activated carbon fibres, carbon aerogels, carbon nanotube, grapheme are been commonly utilized in SCs. Transition metal oxides/hydroxides (TMO/OH) based materials composed of mainly Mo, Co, Ni, V Fe, Ti, and Nb like redox-active transition metals are greatly explored for different applications. Compared to carbon-based materials, TMO/OHs have offered lower resistance, better conductive and enhanced specific capacitance [22]. Furthermore, they have also shown longer operation cycles and excellent specific capacitances. MnO_2_, RuO_2_, Co_3_O_4_, Fe_3_O_4_, NiO, V_2_O_5_, IrO_2_ etc. are the typically used TMOs in SCs studies [23,24,25,26]. Due to having in-between large space of the layers, Co(OH)_2_, Ni(OH)_2_, FeOOH like TMOHs have delivered efficient redox activity which enhanced capacitance of SCs. Likewise various types of conducting polymers (CPs) such as polypyrrole, polythiophene, polyfurane, poly(p-phenylenevinylene), poly(p-phenylene), polyaniline, polyindole, poly 3,4-ethylenedioxythiophene etc. are successfully employed as SC electrodes. The features that make CPs attractive for usage are their capability of withstanding the alteration in redox activity through chemical modification and their structural stability. In addition, to these type of electrode materials their composite or hybrids are also been considered as potential candidates for future exploration [27,28].

Scaling down the electrode materials exhibited additional progress in electrochemical activity of SCs [29]. Modification in size of the similar carbonaceous materials, TMOs, transition metal dichalcogenides etc. are done to convert them into quantum dots (QDs) for better accessibility to the electrolytes which in turn influence the interaction of electrode to electrolytes hence boost up the electrochemical performance. In order, to find and develop well-suited downsized or nanosized materials for SCs, researchers have studied various types of QDs such as carbon, graphene, nickel oxide, tin oxide, niobium pentoxide, hematite, tungsten and molybdenum oxide etc. In upcoming sections, we will try to illustrate each of them with their respective examples along with their benefits and drawbacks. Out of the investigated QDs, carbonaceous QDs are the most repetitively used individually or compositing with other compounds. In 2004 carbon QDs and in 2006 graphene QDs were first introduced and from then on via utilizing modifications or alterations, they are successfully used in different scientific applications. Owing to beneficial characteristics such as, strong luminescence, photobleaching resistance, water solubility, robust chemical inertness, easy & low cost preparation method, biocompatibility and very less toxic nature etc. carbon QDs are seemed to be an fascinating compound which have shown an comprehensive behavior in various scientific fields especially in energy storage ones [30]. Compared to carbon QDs, graphene QDs have almost negligible differences in characteristics. The major distinguishing factor about graphene QDs are their superior crystalline structure, as the GQDs possess graphene lattice inside it in smaller size. The GQDs are found with novel properties due to its quantum confinement effect or large edge effect [31]. However, GQD is a zero-dimensional material by converting two-dimensional graphene. This resulted in quantum confinement and edge effects, which the crystal boundary significantly modifies electron distribution due to the reduced dimension of the crystal to nanometer scale. This also provides GQDs different properties than graphene like non-zero band gap etc.

Apart from employing QDs individually, addition of polymers or specifically conducting polymers have exhibited promising improvements. For instance, the organic polymer composite with quantum dots (PQDs) with different morphologies can be required in solar cells, light emitting diodes, biological sensors, memory devices or waveguide applications etc. [32,33,34,35]. Nanostructuring conjugated polymers with inorganic QDs forming core-shell PQDs nanofibers can be incorporated in solar cells for high efficient modern electronics [33]. Similarly, luminescent colloidal QDs like CdSe(core)/ZnS/CdS/ZnS(shell) QDs compositing with organic ligands polyfluorene and its derivatives are implemented in hybrid light emitting diodes for emitting white light [32]. Interestingly, the study revealed that the degree of emission of the PQDs composites depends on the chemical structure and ratio of polymer and QDs in the composite. The synthesis method for the structure can control the emission spectrum close to the spectrum of daylight and stability. Where the amount of polymer and QDs in PQDs can regulate the conductivity and energy transfer properties, such property can be promising electroluminescent for LED or electrodes for energy storage applications etc.

In general, conductive polymers are preferred in many studies due to their features such as excellent specific capacitance, adequate surface area, better charge conductivity, active reversible reaction, better intrinsic flexibility, low-cost and facile preparation routes. Although lower cycle life and low mechanical stability has put few performances restriction and to overcome that amalgamation of QDs is one of the lucrative options. The π–π stacking interaction, van der Waals interaction, or electrostatic interaction are the three noncovalent approaches between the carbonaceous QDs and conductive polymer through which the electron transfer can be improved and hence the capacitive behaviour. So, here we are going to illustrate few examples regarding these composites. For instance, Devadas and Imae synthesized two separate polymer/carbon dots composites where two different polymer compounds were used which are polyaniline (PANI) and polypyrrole (PPY) [35]. Compared to the individual performance of the carbon dots or original polymers, the composites gave superior outcome in terms of specific capacitances. The carbon dots/PANI exhibited specific capacitances of 529 F g^−1^ whereas the carbon dots/PPY depicted of 676 F g^−1^. The author clearly mentioned that due to the combination of the polymer and carbon dots, the electron transport and ionic motion got positively impacted and hence faster redox reactions as well as excellent specific capacitance is been achieved. Mittal and the group synthesized carbon QDs/PPY nanocomposite which exhibited specific capacitance of 240 mF g^−1^ which was almost 33% more compared to the capacitance recorded for PPy electrode (180 mF g^−1^) [36]. The author concluded that the interfacial charge transfer between the PPy and carbon QDs are responsible for its enhanced electrochemical activity. In another study, combination of graphene QDs, PANI and polyacrylonitrile (PAN) seemed to be able to form mechanically stable and optically active composite film which exhibited specific capacitance of 589.2 F g^−1^ [37]. The as-prepared composite has shown excellent optical absorbance at ~270 nm and outstanding electrical conductivity (2.362 × 10^−6^ S m^−1^). However, we will try to emphasize more with their examples in next sections which will help in closely understanding both of their capacities as well as we will discuss other employed PQDs in SCs and their future prospects.

## 3. Synthesis of Quantum Dots, Polymer, and Nanocomposites

### 3.1. Electrochemical Process

Apart from hydrothermal and microwave irradiated QDs preparation, electrochemical process is another well known and widely studied technique. This process is one of the major categories of top down synthesis approach. Unlike other synthesis method, this process is been reported to be examined from several minute reaction duration to several days. However, few studies have mentioned its swift reaction period and lesser temperature requirement which can easily lead to scaling up of the material production [38]. For an instance, Li et al. prepared 4 nm, uniform, and monodispersed CQDs with strong and stable photoluminescence via employing graphite rods as both cathode and anode in NaOH/EtOH electrolyte where the author mentioned that production rate of CQDs was around 10 mg per hour for each processing setup [38]. Similar to CQDs, GQDs also can be synthesized in facile conditions. Li et al. utilized graphene sheet as an electrode with phosphate buffer solution to prepare water soluble GQDs of 3–5 nm size [39]. So, this process have appropriately exhibited electrochemical oxidation of the carbon precursors to synthesize CQDs as well as appropriate pathways for GQDs or doped GQDs [40,41]. By employing single-stage facile alternating voltage electrochemical technique, preparation of NiO quantum dots/graphene composite was completed where both Ni flake and Graphite rod were exfoliated via using alternating voltage of 5 V and NaOH (2 M) electrolyte was used [42]. With using Ni flake and graphite rod as electrodes, the as-prepared NiO quantum dots were uniformly dispersed on the surface of graphene and an enhanced electrochemical activity was achieved using the prepared composite electrode material. Preparation of WS_2_ and MoS_2_ via electrochemical technique is also been explored by many researchers [43]. Valappil et al. prepared WS_2_ QDs by synergistic effect of Lithium Perchlorate intercalation and propylene carbonate used as electrolyte [44]. Potential of 2 V was applied with the described electrochemical setup. The as-prepared WS_2_ QDs have average size of 3 nm with few-layers and exhibited PL emission (PLQY = 5%). Similarly, MoS_2_ QDs (size of 2.5 to 6 nm) were prepared via one step electrochemical method from its bulk material along with using diluted aqueous ionic liquid solutions of 1-butyl-3-methylimidazolium chloride and bis-trifluoromethylsulphonylimide [45]. As-prepared MoS_2_ QDs exhibited excitation dependent luminescence, which could be further enhanced via surface passivation.

**Figure 1:**
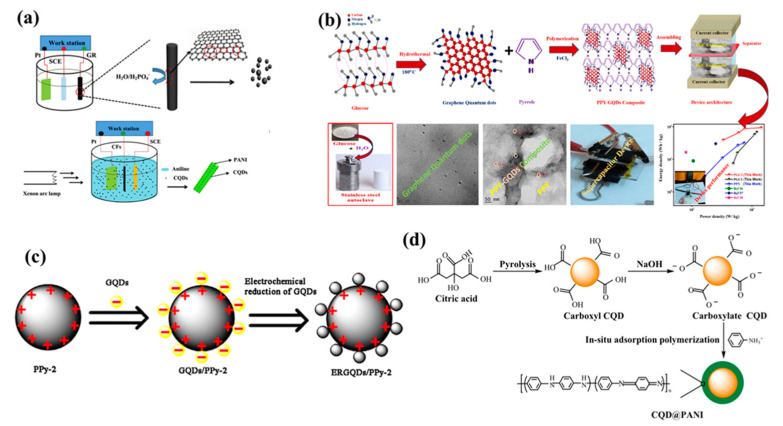
(**a**) Schematic illustrations of electrochemical fabrication of CQDs and CQDs-PANI/CF(LI), (**b**) A schematic diagram represents the synthesis mechanism of GQDs and PPY-GQDs composite fabricated as a supercapacitor device, (**c**) Schematic illustration showing the preparation of ERGQDs/PPy-2, (**d**) Schematic diagram of the preparation procedure of CQD@PANI nanoparticles. Reprinted with permission from: Ref. [47]. Copyright 2016 Elsevier; Ref. [48]. Copyright 2020 Elsevier; Ref. [51]. Copyright 2021 Elsevier; Ref. [52]. Copyright 2019 ACS.

So, this process have successfully shown advantages like enhanced production yield, flexible usage of electrolytes, superior stability without any additive binders during synthesis, efficient exfoliation via regulating the potential or current [43,46]. More importantly, size controllability is another crucial aspect which can be achieved with this process by altering temperature of electrolyte [40]. However, electrochemical process involves graphite or expensive carbon precursors for synthesis which raises overall production costs. Also, unnecessary attached residual additives on the surface of QDs have restricted efficiency of this method. Zhao et al. reported carbon quantum dots-polyaniline hybrid material, the PANI was grown on carbon fiber substrate resulting in a interconnected network structure as shown in Figure 1a [47]. Vandana et al. reported quantum dots dispersed on polymers synthesized through a facile hydrothermal method using glucose as source for quantum dots as shown in Figure 1b [48]. She et al. elaborated on the synthesis of graphene quantum dots and polypyrrole hybrids. The electrochemically reduced graphene was decorated on the polypyrrole sphere as shown in Figure 1c [49]. Li et al. reported core-shell@PANI using citric acid as precursor which follows pyrolysis and NaOH treatment, further the PANI was composited by in situ adsorption polymerization as described in the Figure 1d [50].

### 3.2. Solvothermal/Hydrothermal Process

These synthesis procedures are very frequently employed not only for the synthesis for QDs, but also for different range of materials having various size ranges and configuration. Mostly, it involves single-step procedure in which organic precursors are treated in a closed autoclave to prepare the QDs under high pressure and temperature as per the requirement. Here selection of precursors plays a vital role because the source used may not only contain carbon but may also have doping elements which can impact the synthesized QDs structure. Citric acid, L-cysteine, melamine, hydrazine, Polyethylene glycol-400, (1, 3, 6)-trinitropyrene, hydrazine hydrate are often used as CQD precursors where as bromobenzoic acid, citric acid are the different precursors used in synthesis of GQDs. Other than these precursors CQDs are also been prepared via dehydration of glucose in sulfuric acid and nitric acid [40]. Additionally, the usage of further sulphur and nitrogen doping are observed to make better application outcome of the CQDs or GQDS. For doping purposes, nitrogen (N) and sulphur (S) sources included in the preparation are such as thiourea, urea, hexamethylenetetramine, ethylenediamine and diethyl diethanolamine which are used to synthesize N or S-doped QDs [40]. However, rather than focusing only on one step approach, researchers have adapted two-step synthesis approach to mainly obtain doped QDs with improved structure and applicability [53]. For an example, GQDs are prepared with citric acid taking as precursor with basic condition hydrothermal processes, and then the as-synthesized QDs are blended with hydrazine and heated for several hours to prepared N-doped QDs. Not only CQDs or GQDs, hydrothermal technique is also been used to prepare CeO_2_/Ce_2_O_3_ quantum dots anchored on reduced graphene oxide sheets of different weight fractions where graphene oxide was synthesized initially using natural graphite flakes via employing modified Hummer’s method [54]. Cerium nitrate hexahydrate was the precursor used in this preparation. Similarly, single-step solvothermal technique was also employed to synthesize paper-like layered CeO_2_ quantum dots doped Ni-Co hydroxide nanosheets which was used as electrode in asymmetric supercapacitor [55]. Utilizing citric acid, thiourea and ceria like precursors along with hydrothermal method, N and S co-doped graphene quantum dots were grown on CeO_2_ nanoparticles and this combination of ceria and graphene quantum dots were found to be providing more active surface area, hence enhanced electrochemical activity [56].

Hydrothermal method is also used to prepare CuO quantum dots via using copper (II) acetate monohydrate precursor and additionally single layer graphene was added on the CuO quantum dot surface for better structural stability (unique core-shell structure) and improved performance [57]. Likewise, CuS, SnO_2_, NiCo_2_O_4_ quantum dots were also produced using copper (II) dithiooxamide, stannous chloride dehydrate, nickel acetate tetra-hydrate and cobalt acetate tetra-hydrate precursors respectively [58,59,60]. In order to synthesize WS_2_ and MoS_2_ QDs, solvothermal technique is been broadly used [61]. Precursors like sodium molybdate and cysteine are been used to form MoS_2_ QDs where as sodium tungstate and L-glutathione were used to prepare WS_2_ QDs [62,63]. L-glutathione and cysteine is mainly used as the source for sulfide. However, studies have also mentioned the use of thiocarbamide, dibenzyldisulfide, thiourea as the sulfide source in synthesizing WS_2_ and MoS_2_ QDs [61]. The key benefits of this method are less hazardous, easier to conduct and less expensive. Moreover, controlling and altering the properties as well as composition of the prepared QDs is possible using this method, which are favorable for application purposes.

### 3.3. Microwave Synthesis

**Figure 2:**
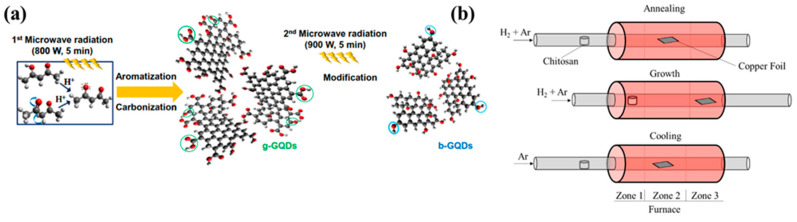
(**a**) Schematic illustration of microwave bottom-up route for GQDs and b-GQDs: green circles mean carboxyl and carbonyl groups and blue circles indicate hydroxyl groups. (**b**) Schematic diagram for the synthesis of N-GQDs. Reprinted with permission from: Ref. [65]. Copyright 2014 Elsevier; Ref. [66]. Copyright 2018 ACS.

Compared to hydrothermal or solvothermal process, microwave irradiated preparation techniques consume less time and required lesser temperature for synthesizing QDs. Via adding water with glucose and ammonia, GQDs are been prepared under microwave irradiation which took only one minute reaction time [62,63]. Another group has prepared similar GQDs in just 5 min under 200 °C using acetylacetone along with water in quartz bowl kept under 800 W power microwave irradiation. Not only because of quicker reaction time and low temperature requirement, but also due to easy reaction route and overall economical factors this technique fits suitable for large scale production of GQDs as suggested by researchers [40,64]. The microwave synthesis of GQDs and its modification by tuning different parameters like power, time temperature etc. have been illustrated in the Figure 2 [65,66]. On top of that, in case of characteristics of CQDs or GQDs can be controlled and altered via adjusting irradiation power and reaction timing or by varying precursors. Although, using the microwave method for production of CQDs are not clearly mentioned in the literature to be completely apt.

### 3.4. Direct Chemical Cutting Process

Broad range of carbon or biomass-derived char are been utilized to produce QDs using direct chemical cutting technique. So, comparably to electrochemical process, this technique engages less initial material cost which reduces total operation expenses. Chemical cutting is been classified into two individual types. First one involves single-stage cutting of carbon materials via applying strong acids and the other category is a two-stage process which is comprised of combinational approach of Hummers method and then chemical reduction process to finally produce QDs. Coal and graphene like carbon sources are frequently used in the single-stage process for synthesis. Chemical cutting is been frequently used to produce both CQDs and GQDs. Using graphene along with nitric acid and sulfuric acid concentrated solution, chemical cutting process can be used to develop GQDs [67]. These acids act as suitable cutter for exfoliating GQDs from various precursors. Afterwards, removal of acid is minutely done by calcining the dried mixture to properly separate the QDs. Apart from conventional precursors, nature derived material like chitosan is been examined and proven to be suitable for preparing doped GQDS [64]. Preparing GQDs using walnut shells along with similar concentrated acid solutions (nitric acid and sulfuric acid) is another novel study which can attract authors to study more different waste materials to produce GQDs via using this process [68,69]. Studies have also examined preparation of CQDs. Precursors like coal, gelatin, are been commonly used to prepare the long chain ligands at the quantum dot surface that can inhibit efficient charge transfer. As, the long chains may increase the pathway of electron transfer, reducing the charge kinetics. The ligands possessing multiple groups on the surface can stability or solubility of composites. The sensitivity and selectivity of the QDs can also be regulated by the ligands attached to the surface. But it depends on the pH medium and salt concentration so regulate the storage ability [70]. Overall, this process can considerably attractive owing to its low cost and simplicity. Yet, few drawbacks were also observed such as longer reaction period, usage of hazardous acids for cutting the precursor layers, difficulties faced during acidic solvent after synthesis etc. These problematic factors not only affect the synthesis but also pose harmful environmental threats.

### 3.5. Hummers Method

Utilization of Hummers method or modified Hummers method is extensively preferred to synthesize GQDs. The process is generally divided into two segments; first one involves preparation of graphene oxide (GO) and then chemical cutting procedure is used to finally prepare GQDs [71,72,73,74]. For an instance, Shen et al. utilized natural graphite powder to synthesize GO via employing modified Hummers method and afterwards heated the as-prepared GO in the nitric acid solution for 24 h to prepare the GQDs [68]. Similarly, Li et al. prepared nitrogen doped GO via the Hummers method and employed chemical cutting process by heating the prepared GO in a concentrated solution of nitric acid and sulfuric acid to finally synthesize N-GQDs [73]. Maintaining similar procedures, coal is also been used along with Hummers method to prepare GQDs. So, it is clearly observed in the reports that usage of Hummers method seems to be more purposeful when it is used jointly with chemical cutting process.

In this section we have discussed the individual synthesis of QDS. Compositing QDs with polymer materials are done by few precise processes such as in situ chemical polymerization, doping methods, solution phase mixing method or simple mixing and filtration methods, direct photo-electrodeposition techniques etc. [18,36,37,75,76,77,78,79]. For example, Devadas and Imae, CTAB, APS along with carbon dots to prepare the composite [80]. Firstly, the CTAB was dissolved in aqueous hydrochloric acid solution and stirred properly. Afterwards, the as-prepared carbon dots solution was added and then Sequentially pyrrole monomer added and whole combination was kept stirring for 30 min with maintaining 0–5 deg. Lastly, APS in aqueous HCl solution was added and filtered using a cellulose acetate membrane filter to obtain final composite product. In another study, Arthisree and Madhuri used the solution phase mixing method to combine PAN dissolved in DMF, PANI and GQD dispersed in DMF with different concentration (PAN: PANI: GQD ¼ 9:1:0, 9:0.5:0.5, 8:1:1, 7:1.5:1.5) and stirred for 12 h at room temperature [41]. Afterwards via drop casting technique, the synthesized composite solutions were spread uniformly on to a glass petri dish and dried for about 76 h at room temperature to finally formed the films to apply for electrochemical studies. Apart from these methods, pyrolysis is another facile technique been successfully used to synthesize core-shell structure of C-QDs coated by PANI [81]. However, for this particular composite preparation these methods need to be further explored to accomplish more compact form and structure.

## 4. Quantum Dots and Polymer Composites in Supercapacitor Applications

**Figure 3:**
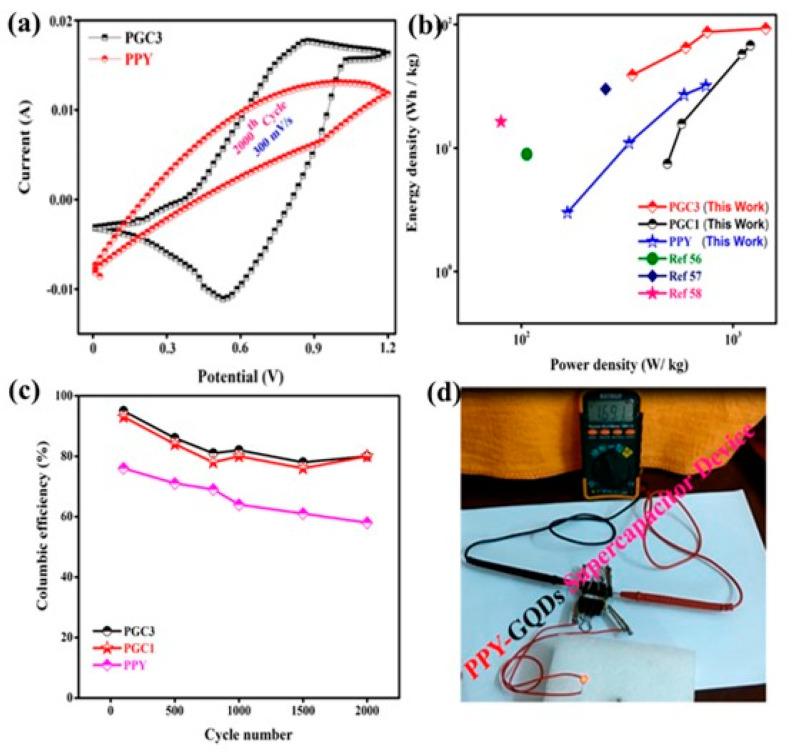
Electrochemical performance of assembled PPY-GQDs supercapacitor device. (**a**) CV curves of the PPY and PGC3 device at a constant scan rate of 300 mV s^−1^, (**b**) Regone plots of PPY, PGC1 and PGC3 composite (**c**) Capacitive retention vs. cycle number of PPY, PGC1 and PGC3, (**d**) A digital image of a supercapacitor device. Reprinted with permission from Ref. [48]. Copyright 2020 Elsevier.

Reducing materials or compounds from its bulk form to QD structure exhibits various advantages such as stable and efficient quantum confinement effects, improved surface area, surface to volume ratio, diffusion length for both electrons and ions, higher concentration of edge atoms or more available edge atoms providing extra active sites, much lesser volume expansion/contraction etc. [54]. These structural benefits augment the electrochemical activity when used as electrode materials in energy storage devices, especially batteries and supercapacitors (SCs). Commonly, improved surface area or higher number of active sites endorses scope of additional reactivity in between the components inside of such kind of devices (batteries and SCs). Therefore, intercalation processes and charge storage kinetics are boosted which in turn increases the capacitance as well as operating potential window [16]. However owing to factors such as agglomeration problem, large number of interfaces etc. QDs faced several electrochemical device performance issues. Because of the van der Waals forces and vast surface energy, QDs are often failed to resist the agglomerating problem. Studies have shown that these issues can be effectively resolved by adding conductive matrix such as MOF with the QDs [82]. Utilization of the hybrid of MOF and QDs enhances the specific capacitance and hence device activity got improved. One of electrochemical performance is given in the Figure 3; displaying the cyclic voltammetry curves, Ragone plot (energy and power density), efficiency of device and demonstration of device with practical application [34].

**Figure 4:**
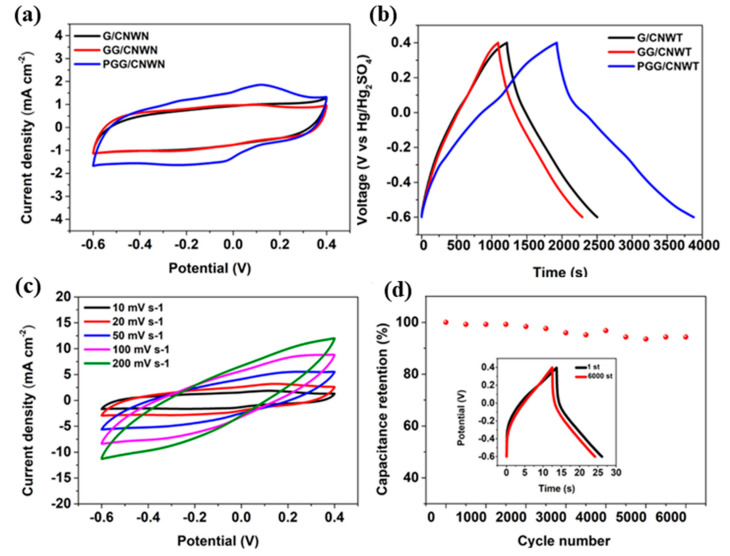
The electrochemical properties comparison of G/CNWT, GG/CNWT and PGG/CNWT: (**a**) CV curves at 10 mV, (**b**) GCD curves at 0.1 mA cm^−2^, (**c**) CV curves of PGG/CNWT at different scan rates, (**d**) Electrochemical stabilities of PGG/CNWT at a current density of 5 mA cm^−2^ for 6000 cycles. Reprinted with permission from Ref. [88]. Copyright 2020 Elsevier.

Apart from the generalized issues, specific issues for specific QDs are been observed in various studies. For instance, ceria QDs are found to be inadequately conductive, so rather than using them individually for SCs, they are necessarily been composited with conductive materials to achieve appropriate electrochemical activities [54]. N. Chakrabarty et al. prepared hybrid composite via mixing reduced graphene oxide (rGO) and ceria which formed conducting pathways and ensures easy movement of charges in the fabricated devices. Interestingly addition of rGO not only resolved the conductivity issues but also prevented the QDs from agglomeration. Similar to ceria, Nb_2_O_5_ QDs have also exhibited conductivity and aggregation issues. In this case, coating these QDs with biomass derived nitrogen-rich carbon have alleviated the drawbacks [82]. Additionally, to counter volume expansion issues, annealing at higher temperature during synthesis was also recommended in the study for even distribution of QDs in nitrogen-rich carbon. Few other problems are also been observed such as restacking problems, achieving uniform QDs with narrow size distributions etc. Reports have further mentioned that such problems can be reduced to some extent by forming composite materials or capping via employing organic groups [43]. Dinari et al. reported PANI/graphene quantum dots as electrode material for supercapacitor using citric acid as carbon source and thiourea as N, S source for heteroatom doping, the composite was prepared by a in situ electrochemical polymerization. The resulting electrode material exhibited superior electrochemical performance than that of PANI [83]. Mehare et al. reported sucrose derived quantum dots composited with polyaniline through electrodeposition. The as-prepared material showed a high specific capacitance of 1512.4 F g^−1^ at 1 A g^−1^ in a three-electrode setup with 1 M H_2_SO_4_ as electrolyte. The assembled asymmetric supercapacitor shows a high specific capacitance of 295 F g^−1^ at 1 A g^−1^. The device showed a remarkable energy and power densities of 40.86 Wh kg^−1^ and 2000 W kg^−1^ [84]. Kumar et al. PANI and carbon quantum dots composite material derived from polyethylene glycol as source for quantum dots, the nanocomposite was synthesized by chemical oxidation. The assembled device showed a high specific capacitance of 161.3 mF cm^−2^ with a good cycling stability even after 5000 cycles [85]. Jian et al. reported electrochemical synthesis of graphene quantum dots composited with polymers, the as-prepared material CQDs/PPy composite ASSSs showed outstanding electrochemical performance with the areal capacitance 315 mF cm^−2^ (corresponding to specific capacitance of 308 F g^−1^) at a current density of 0.2 mA cm^−2^ and the stability had long cycle life with 85.7% capacitance retention excellent performance till 2000 cycles [86]. Shao et al. reported PANI/graphene quantum dots/graphene co-coated on the commercial compressed non-woven towel through a repeated dyeing and drying method, the material exhibited a high specific capacitance of 195 mF cm^−2^ at 0.1 mA cm^−2^ with a high stability of 96.5% retention after 6000 cycles [87]. Similarly, Figure 4 represents the CV and charge/discharge curves of the electrodes based on PANI composites with graphene QDs. The Figure 4d shows the cycle life of the electrodes for 6000 charge/discharge cycles, where it is found to be quite good stable. Thus, this suggests compositing polymer with QDs can be potential electrode to acquire high electrochemical performances.

## 5. Electrolytes Used for Polymer Composite QDs Electrodes for Supercapacitor

Similar to the electrodes, electrolyte is essential component of the SCs. Electrode material having more accessibility of electrolyte ions results in superior electrochemical activity, so that is why enhanced contact area and wettability between the electrolyte and electrode is required to promote the redox reactions. It is very important to understand that the electrostatic charge separation between electrode-electrolyte is used for harvesting energy in SCs. Additionally, it is also been observed that the functional-containing groups of electrodes are beneficial for faradic redox reactions due to the robust contact with electrolyte ions. So, selecting optimized electrolyte is important as their co-ordination with specific electrode material will exhibit better outcome. Electrochemical parameters like, energy density, power density, cycle life partly depends on the electrolytes employed to fabricate SCs. Moreover, electrolytes must also be selected considering the safety aspects also as they should not react with electrode or binder material and destabilize the system. To design electrolytes in a very specific way few factors must be taken into consideration such as; ionic mobility and conductivity, salt concentration, viscosity, electrochemical and thermal stability [89]. In recent studies various types of electrolytes such as solid and quasi-solid-state electrolytes, organic electrolytes, redox-active electrolytes, ionic liquid electrolytes, aqueous electrolytes are been extensively explored [90]. Different electrolytes are recommended for different setups, for example asymmetric and flexible supercapacitor cell are found to be working in a superior way when an aqueous electrolyte is employed [91,92]. The ultra-small size of QDs greatly reduces the diffusion pathway of electrolyte ions in the bulk phase, which further lead to faster ion/charge transfer [93]. Commonly electrodes prepared with QDs have preferably used electrolytes like NaOH, H_2_SO_4_, KOH, Na_2_SO_4_ [94,95,96]. Apart from these, utilization of organic electrolyte is also been investigated. S. Liu et al. [97] employed LiPF_6_ in ethylene carbonate/dimethyl carbonate where Nb_2_O_5_ QDs embedded in nitrogen-doped porous carbon derived from ZIF-8 dodecahedrons was used as electrode. In another study, the similar organic electrolyte used where Nb_2_O_5_ QD coated with heteroatom biomass carbon was used as electrode [98]. It was clearly identified in the study that heteroatom carbon can uphold the stability of solid-liquid interface between electrolyte and electrode which in turn useful in steady transport of charges and ions and hence suitable electrochemical performance achieved. Interestingly studies have shown that compared to single oxides, binary metal oxide performs in a better way, as in many cases restricted transport among the electrolyte and electroactive material has been observed [99]. Few studies have reported the utilization of PVA/KOH and PVA/H_2_SO_4_, PVA/H_3_PO_4_ gel electrolyte which exhibited high capacitive activity [100,101].

## 6. Discussing Pros and Cons on Polymer Composite QDs as Electrode in Supercapacitors

As we discussed in the previous sections it seemed very clear that the enhanced surface area of QDs is very useful in improving the electrochemical performance of SCs. Moreover, other identified benefits were higher concentration of edge atoms, high density of active sites, larger surface to volume ratio, which makes the charge transport inside the device more precise and favorable, and as a result, higher efficiency was achieved. In this review, we have already mentioned individual advantages and drawbacks of PQDs through the examples of various recent studies and discussed their respective outcomes to portray a clear picture of the suitability of PQDs in SCs. During the discussion, we have certainly tried to identify few aspects which requires consideration to develop more efficacious SC setup using different PQDs and to finally to increase the possibility of their commercial or large-scale usage purpose. The studies need to be more focused on the charge storage mechanism both theoretically as well as experimentally for the newly reported polymer composite/hybrids of QDs. Additionally, the studies claimed to be deriving carbon based-QDs from biomass or waste biomass sources must present, which on addition to polymer can be eco-friendly. The detailed analysis of their electrochemical analysis comparing them with their non-natural counterparts as it will be useful to differentiate their impacts in a better way. Thus, making the energy storage devices sustainable and clean energy. Synthesis procedures of PQDs as well as their functionalization routes also needs to be improved to make the synthesis techniques more facile, low-cost and efficient as it will impact the overall expanse of fabrication and their electrochemical activity. But importantly with maintaining the overall cost (starting from synthesis to device fabrication) the electrochemical parameters such as specific capacitance, cycling stability, energy density, and power density are also needed to be monitored as balancing these factors will finally result into an efficient device. So, in order to develop further crucial enhancements, the discussed aspects must be taken account of. However, with the ongoing research possibly we may be able to encounter more important facts about the engagement of PQDs in SCs application which probably may able to guide the upcoming future technologies more sustainable and accessible.

## 7. Future Perspective of Polymer Composite QDs Electrodes in Supercapacitors

Besides having application in photovoltaic devices, bio-imaging, chemical and metal ion sensors etc, PQDs have effectively shown their promising usage in electrochemical energy storage and conversion devices which we have attempted to discuss elaborately in this chapter. Owing to their unique chemical and physical characteristics along with features like high selectivity to special molecules, adequate liquid dispersibility, facile surface functionalization, QDs became a relevant choice to utilize in developing suitable components of battery and supercapacitor like devices. Oxide QDs like CeO_2_, Cu_2_O, SnO_2_ etc. and sulphide QDs CuS, MoS_2_, NiS, WS_2_ etc. have exhibited great application scopes with using them in different supercapacitor applications. Mxene QDs are also gaining severe consideration due to their excellent electronic properties; although still need vast surveying for better optimizations. Other than that, recent developments have focused on compositing these polymers or QDs with other fitting compounds such as carbon nanotubes, multi-walled carbon nanotubes, reduced graphene oxide in order to raise the device performances like cycle stability, capacitive activity etc. Table 1, summarizes the graphene composited polymer electrodes comparison from the table we can observe that PANI and PPy has been widely explored and they hold good electrochemical properties as electrode for supercapacitor. 

Even though the performance figures are high, stability of polymers has been still less even after compositing with quantum dots. However, harmonizing between their efficiency and eco-friendly aspect is still underway to make them compatible for larger scale deployment. Currently, approaches like green synthesis methods, hybridizing QDs with conducting polymers like PANI, Polypyrrole, etc. are also getting significant attention as they not only improving the performance of the electrochemical device and their nature companioning status by avoiding usage of toxic chemicals but also helping in cost cutting in overall preparation process. Apart from those, bio-derived natural polymers like cellulose, chitin, or chitosan are also be excellent options to combine with QDs to quest for hopeful enhancements in device outcomes.

## 8. Applications of Biomass Derived Materials and Plastic Materials for Energy Storage Devices

Manufacturing of energy storage materials from biomass-derived and plastic materials will generate less waste leading to the world’s circular economy. Pyrolysis of carbon-neutral biomasses yield biochar, bio-oil and syngas etc. Biochar can be utilized to catalyze pyrolysis, to create syngas and trans-esterification, while particles made of bio-char are used for machineries, e.g., fuel cells, super capacitors, and batteries. Nayak and his group [116] have discussed in detail the use of biochar and biochar based materials as the origin of power, as catalyst to produce energy, for trans-esterification, for pyrolysis, generation of syngas, in case of oxygen electro catalyst, to create fuel cell, to make super capacitors, or even to generate batteries.

Carbon material derived from biomass are popular as electrodes into energy storage devices due to their variable physical and chemical characteristics, high impact on environment socioeconomic cost. Zhang and his co-workers [117] have discussed in detail how to control the properties of biomass derived materials for the production of electrodes of energy storage devices including electro-chemical supercapacitors, lithium-ion batteries and many more. To store energy we have been using heavy metal batteries. In recent times, organic batteries have become very good alternative for the energy storage device, moving towards sustainable systems. In recent days electrodes in batteries are generated from any carbonized or non-carbonized biomasses; that means all types of waste biomass can be carbonized and can be utilized to prepare anodes in lithium or sodium ion batteries or to prepare cathodes in metal-sulfur or metal-oxygen batteries [118].

A group from university of california, riverside has been working for many years to develop better quality energy storage particles from renewable origins, e.g., glass bottles, beach sand, Silly Putty, and portabella mushrooms to reduce plastic pollution and hasten the transition to 100% clean energy [119]. They have developed a low cost method to prepare batteries made of nanomaterials from waste plastics.

The polymeric or plastic materials with high conductivity and larger redox active capacitance can be used as pseudocapacitive materials. Fang and his group [120] have discussed the application of polymers, plastic materials and their combinations for use as electrodes and electrolytes in high-performing supercapacitors and lithium-ion batteries. They also mentioned how the power and energy densities of supercapacitors and lithium ion batteries can be developed from the polymeric materials.

## 9. Conclusions

In last few decades energy conversion and storage devices have immensely been progressed which impacted the next generation technologies in a prominent way. Utilization of batteries, SCs are becoming crucial in new edge electronics and hopefully in upcoming days we will be relying more on them. SCs are found to be very reliable for using in different real-time applications. They are proven appropriate candidate for next generation energy-oriented technologies like solar systems, faster trains, electric vehicles, and wind power equipment, owing to their advantageous properties like excellent power density, longer cycle life, involvement of lesser cost etc. However, studies are still trying to analyse, and improve their properties to make their usage more efficient. In this way SCs hand in hand with batteries will able to resolve many energy issues and able to provide a substantial alternative for energy fuels. In this review, we have concentrated our discussion on different types of quantum dots (QDs) and polymer or nanocomposites employed for SCs with illustrating their individual performance. Impactful utilization of QDs have actually triggered to achieve more efficiency in the performance of SCs. Although not only in case of the SCs, QDs have also contributed excellently in other applications such as for battery electrode materials, drug delivery, biosensor, bioimaging, electrochemical, UV, sensor etc. In this review, we have specifically focused our discussion on electrode materials based on quantum dots, polymers and their composites reported till date. The existing studies helped in unrevealing various important insights about their unique properties and minute observations from their utilization in numerous SC configuration was also helpful to understand their functioning. By observing these studies, it was been clearly identified that the polymer nanocomposite with carbon based QDs are the ones mostly preferred in studies as they have depicted adequate electrochemical performances in terms of power density and cycle life. However, other types of QDs such as transition metal oxide QDs, transition metal dichalcogenide QDs, and polymer QDs are also found to be effectively workable owing to their stable cyclic performances, unique layered structural benefits, and enhanced pathways for mass/ion transfer. Currently many research ideas are seemed to be inclined towards converting the biomass or waste materials to prepare suitable materials for applying in components of SCs or battery like devices. Few studies similar to that are already been reported involving of PQDs as well. Hopefully, more studies will engage analogous efforts as with these approaches, we will not only be able to reduce the overall cost of the fabricated SCs but also, we will able to develop ways to make appropriate utilization of waste materials for energy application rather than simply landfilling it. Finally, by observing the existing and ongoing evaluation and assessments of the PQDs, it is easy to interpret that in near future we will be able to encounter much precise utilization and advancement of PQDs in SC devices which may possibly help in establishing large scale industries using the same.

## Figures and Tables

**Table 1:** Polymer composites with quantum dots based-electrodes for supercapacitors application.

Electrode	Electrolyte	Specific Capacitance	Retention Rate (Cycles)	Energy Density	Power Density	Ref.
CQDs/PPy PPY-GQDs	PVA-LiCl 1 M NaCl	308 F g^−1^ 647.54 F g^−1^	85.7 % (2000) 91.7% (2000 cycles)	NA 93 Wh kg^−1^	NA 1430 W kg^−1^	[86] [102]
GQDs/3DG	1 M KOH	242 F g^−1^	93% (10,000)	NA	NA	[103]
S-CQD/PANI	1 M H_2_SO_4_	295 F g^−1^	80% (1000)	40.86 Wh kg^−1^	2000 W kg^−1^	[104]
CQDs/PPy-NW	1.0 M KCl	306 F g^−1^	85.2% (5000)	NA	NA	[105]
PANI/S,N:G QDs	2 M KOH	2524 F g^−1^	100% (1000)	47.78 Wh kg^−1^	2250 W kg^−1^	[106]
GQDs//PANI	H_3_PO_4_–PVA	667.5 μF cm^−2^	85.6% (1500)	0.093 μ Wh cm^−2^	7.52 μ W cm^−2^	[107]
CQDs-PANI	H_2_SO_4_–PVA-EG	738.3 F g^−1^	78.0% (1000)	33.8 μ Wh cm^−2^	0.3 mW cm^−2^	[108]
GQDP	0.5 M H_2_SO_4_	1044 F g^−1^	80.1% (3000)	117.45 Wh kg^−1^	448.8 W kg^−1^	[109]
PPy/CQDs	1M KCl	1073 F g^−1^	62 % (2000)	70.22 Wh kg^−1^	3060 W kg^−1^	[110]
PVA-GQD/PEDO	1 M H_2_SO_4_	291.86 F g^−1^	98& (1000)	16.95 Wh kg^−1^	984.4 W kg^−1^	[111]
CQDs/PPy-Fe	H_2_SO_4_– PVA	317 F g^−1^	94.6% (2000)	52 Wh kg^−1^	900 W kg^−1^	[112]
S,N-GQD/PANI	1 M H_2_SO_4_	645 F g^−1^	90% (1000)	17.25 Wh kg^−1^	500 W kg^−1^	[113]
ERGQDs/PPy	1 M H_2_SO_4_	418 F g^−1^	86% (1000)	NA	NA	[114]
MnO_2_/PANI/rGO QDs	1 M H_2_SO_4_	423 F g^−1^	85% (2000)	34.47 Wh kg^−1^	640 W kg^−1^	[115]

## Data Availability

The data presented in this study are available upon request from the corresponding author.
